# Draft Genome Sequence of the Endosymbiont “*Candidatus* Ruthia magnifica” UCD-CM (Phylum *Proteobacteria*)

**DOI:** 10.1128/genomeA.00717-14

**Published:** 2014-07-17

**Authors:** Ruth D. Lee, Guillaume Jospin, David A. Coil, Jonathan A. Eisen

**Affiliations:** a Genome Center, University of California Davis, Davis, California, USA; b Department of Evolution and Ecology, Department of Medical Microbiology and Immunology, University of California Davis, Davis, California, USA

## Abstract

Here, we present the draft genome of the endosymbiont “*Candidatus* Ruthia magnifica” UCD-CM, a member of the phylum *Proteobacteria*, found from the gills of a deep-sea giant clam, *Calyptogena magnifica*. The assembly consists of 1,160,249 bp contained in 18 contigs.

## GENOME ANNOUNCEMENT

The gammaproteobacterial endosymbiont “*Candidatus* Ruthia magnifica” was previously found to be an obligate, intracellular autotroph in one species of giant clam, *Calyptogena magnifica* (1–3). “*Candidatus* Ruthia magnifica” possesses the ability to fix carbon for its host, although the specific biochemical mechanisms of this ability remain elusive (4, 5).

*Calyptogena magnifica* was collected from a 28 May 2002 deep-sea exploration of a hydrothermal vent located in the Galápagos Rift, via the submersible, DSV *Alvin*, dive 3790 (6). Gill tissue was dissected and frozen in liquid nitrogen. Genomic DNA was extracted as previously described for environmental samples (7). Illumina paired-end libraries were made using a modified version of the Nextera kit by Illumina but with homegrown transposase.

A total of 3,587,578 paired-end reads were generated on an Illumina MiSeq, at a read length of 160 bp. Quality trimming and error correction of the reads resulted in 3,500,962 high-quality reads. All sequence processing and assembly was performed using the A5 assembly pipeline (8). This pipeline automates the processes of error correction, data cleaning, scaffolding, contig assembly, and quality control. The resulting assembly produced 17,632 contigs, with an *N*_50_ of 591. Screening the contigs using NCBI BLASTx against the NCBI’s nonredundant GenBank database showed a preponderance of non “*Candidatus* Ruthia magnifica” (human, *Escherichia coli*, or other) hits. A consequent BLAST filter against a “*Candidatus* Ruthia magnifica” reference database, however, identified 18 of these contigs as “*Candidatus* Ruthia magnifica” and increased the genome *N*_50_ to 105,440. The resulting genome consisted of 1,160,249 bp, with a GC content of 34% and an overall coverage estimate of 19×. Scaffolds were verified by mapping error-corrected reads to the assembly using the Burrows-Wheeler Aligner (BWA) (9). Completeness of the genome was assessed using PhyloSift software (10), which searches for a list of 37 highly conserved, single-copy marker genes (11), of which all 37 were found in this assembly.

Automated annotation was performed using the RAST server (12). “*Candidatus*” sp. strain UCD-CM contains 1,215 predicted protein-coding genes and 40 predicted noncoding RNAs.

### Nucleotide sequence accession numbers.

This whole-genome shotgun project has been deposited in DDBJ/EMBL/GenBank under the accession number JARW00000000. The version described in this paper is the first version, JARW01000000.
